# Exploring Integrative Analysis Using the BioMedical Evidence Graph

**DOI:** 10.1200/CCI.19.00110

**Published:** 2020-02-25

**Authors:** Adam Struck, Brian Walsh, Alexander Buchanan, Jordan A. Lee, Ryan Spangler, Joshua M. Stuart, Kyle Ellrott

**Affiliations:** ^1^ Biomedical Engineering, Oregon Health and Science University, Portland OR; ^2^ Biomolecular Engineering Department, University of California, Santa Cruz, Santa Cruz, CA; ^3^ University of California Santa Cruz Genomics Institute, University of California, Santa Cruz Santa Cruz, CA

## Abstract

**PURPOSE:**

The analysis of cancer biology data involves extremely heterogeneous data sets, including information from RNA sequencing, genome-wide copy number, DNA methylation data reporting on epigenetic regulation, somatic mutations from whole-exome or whole-genome analyses, pathology estimates from imaging sections or subtyping, drug response or other treatment outcomes, and various other clinical and phenotypic measurements. Bringing these different resources into a common framework, with a data model that allows for complex relationships as well as dense vectors of features, will unlock integrated data set analysis.

**METHODS:**

We introduce the BioMedical Evidence Graph (BMEG), a graph database and query engine for discovery and analysis of cancer biology. The BMEG is unique from other biologic data graphs in that sample-level molecular and clinical information is connected to reference knowledge bases. It combines gene expression and mutation data with drug-response experiments, pathway information databases, and literature-derived associations.

**RESULTS:**

The construction of the BMEG has resulted in a graph containing > 41 million vertices and 57 million edges. The BMEG system provides a graph query–based application programming interface to enable analysis, with client code available for Python, Javascript, and R, and a server online at bmeg.io. Using this system, we have demonstrated several forms of cross–data set analysis to show the utility of the system.

**CONCLUSION:**

The BMEG is an evolving resource dedicated to enabling integrative analysis. We have demonstrated queries on the system that illustrate mutation significance analysis, drug-response machine learning, patient-level knowledge-base queries, and pathway level analysis. We have compared the resulting graph to other available integrated graph systems and demonstrated the former is unique in the scale of the graph and the type of data it makes available.

## INTRODUCTION

Biological data produced by large-scale projects now routinely reach petabyte levels thanks to major advances in sequencing and imaging. With multiple profiling methods, platforms, versions, formats, and pipelines, a major unaddressed issue is querying across the increasingly heterogeneous data. When faced with the substantial labor and computation costs, researchers may use outdated and/or only a fraction of publicly available data.

Graph databases are useful tools for integrating complex and interconnected data.^1-3^ In the commercial sector, several major data aggregators have successfully used graph databases to integrate heterogeneous data. Facebook (Menlo Park, CA) uses the “Social Graph”^4^ to represent the connections between people and their information, whereas Google’s search engine (Alphabet, Mountain View, CA) uses Google’s “Knowledge Graph” to connect various facts about different subjects. On the basis of these observations, we have built the BioMedical Evidence Graph (BMEG) to allow for complex integration and analysis of heterogeneous biological data.

The BMEG was created by importing several cancer-related resources and transforming them into a coherent graph representation. These resources include patient and sample information, mutations, gene expression, drug response data, genomic annotations, and literature-based analysis (Appendix Table A1). The BMEG contains data on 15,000 patients, 52,000 samples, 6.8 million alleles, 640,000 drug-response experiments, and 50,000 literature-derived genotype-to-phenotype associations.

We describe a resource that enables analysts to quickly access data from multiple sources and perform queries that integrate them with clear graph-based semantics. Independently, a researcher would need to download hundreds of files, write multiple file parsers, develop an integrated data model, map identifier systems, and normalize analytical results. The BMEG centralizes that work and makes it searchable using a full-feature query engine. To enable analysis and machine learning, the BMEG includes high-quality feature-extraction methods applied consistently to all samples. This includes the results provided by the best methods of somatic variant calling and RNA-sequencing analysis for cancer genomic and transcriptomic data sets. We used open challenges to create leaderboards of the best methods from all of those submitted by the community. We then participated in the development of open standards to enable the exchange of genomic associations from cancer knowledge bases. Finally, we implemented computational integrity checks and unit tests for each of the data import modules.

**CONTEXT**

**Key Objective**
Can a graph database help researchers access multiple integrated data sets for cancer omics analysis?
**Knowledge Generated**
We have developed and tested an integrated graph database, BioMedical Evidence Graph, that connects patient sample data, cell-line drug-response data, and multiple knowledge bases. Simple queries to this system allowed cross–data set analysis in seconds when the same questions would have required days or weeks of manual effort otherwise.
**Relevance**
Analysis of cancer systems biology data requires a large variety of different kinds of data. The BioMedical Evidence Graph system provides a uniform interface for data interrogation that will make it easier to pose a variety of clinically relevant queries.

## METHODS

### Graph Schema

**FIG 1.**
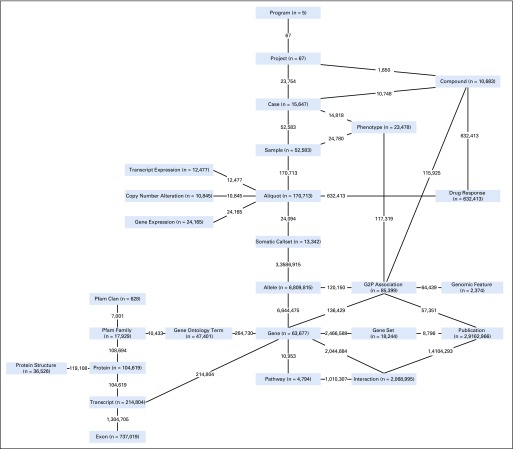
The BioMedical Evidence Graph schema showing the vertex types and connections of the graph. Numbers on vertices represent the total instances of a specific type defined by the vertex (eg, the Gene vertex includes 63,677 distinct protein-coding, microRNAs, and other gene entries); numbers on an edge connecting two vertices represent the total connections between any instance of the first vertex to any instance of the second vertex (eg, there are 214,804 connections from transcripts to the genes that encode them). Pfam, protein families.

At the core of BMEG’s metadata is a tree representing the organization of all the different data elements (Fig 1). *Program* node represents the root of the tree, defining a cohort of samples studied by a consortium. For example, The Cancer Genome Atlas (TCGA; National Cancer Institute, Bethesda, MD) is one such “program” and cohorts for different tumor types can be selected using the program’s child node called “Project.” Each tumor type is then populated by a number of *Case* nodes, which in turn have multiple *Sample* nodes, which can then be subdivided into a number of *Aliquot* nodes. The BMEG schema builds on this base structure to include data from a number of additional sources including: (1) genome reference, (2) gene and pathway annotations, (3) somatic variants, (4) gene expression data, and (5) knowledge bases.

### Data Sources

Initial data sources (Appendix Table A1) for the BMEG were centered on large cohorts of patient-derived samples, with DNA and RNA profiling, cell lines with drug-response data, and literature-derived drug-phenotype associations. The goal was to provide uniform input data for analysis and machine learning.

### RNA-Sequencing Data

To identify the best methods for RNA analysis, we launched the somatic mutation calling–RNA challenge, which benchmarked isoform quantification methods to prioritize the methods used for processing data that would be ingested into the BMEG. For example, for transcript abundances we used results from Kallisto (Patcher Lab, University of California, Berkeley, Berkeley, CA), a top contending method in the somatic mutation calling–RNA challenge, to process the TCGA and Cancer Cell Line Encyclopedia (CCLE)^5^ data sets. In addition, the Genotype-Tissue Expression project (National Institutes of Health, Bethesda, MD)^6^ provided gene-level, transcript-per-million-mapped reads estimates for normal tissues that could be contrasted with tumors. Combinations of these resources provide 36,000 vertices to the BMEG graph.

### TCGA Metadata

The Genomic Data Commons (GDC; National Cancer Institute, Bethesda, MD) created a data system to track the clinical and administrative meta-data of the TCGA samples and files. We used their web application programming interface (API) to obtain TCGA patient and sample metadata for the evidence graph.

### TCGA Genomic Data

To determine the best methods for somatic mutation calling, we partnered with the Dialogue on Reverse Engineering Assessment of Methods (DREAM) consortium, Sage BioNetworks (Seattle, WA), and the Ontario Institute for Cancer Research to launch the International Cancer Genome Consortium–TCGA Somatic Mutation Calling challenge.^7^ Many methods evaluated by this effort were incorporated into pipelines that would be deployed on the TCGA’s 10,000 exomes as part of the Multi-Center Mutation Calling in Multiple Cancers (MC3) project.^8^ The MC3 adds 10,000 vertices that connect to 3 million alleles (2.6 million distinct alleles) in the graph. For the set of copy-number alteration events, we used the Gistic2^9^ data from the Broad Institute’s Firehose system (Massachusetts Institute of Technology, Cambridge, MA).

### Cell-Line Drug-Response Data

Cell-line clinical attributes and drug-response data has been collated by the DepMap (Broad Institute)^10^ and Pharmacodb (BHKLAB, Princess Margaret Cancer Centre – University Health Network, Toronto, ON, Canada)^11^ projects, respectively. This includes response curves, half maximal inhibitory concentration and half maximal effective concentration (EC_50_) scores from CCLE,^12^ Cancer Therapeutics Response Portal (CTRP)^13,14^ and Genomics of Drug Sensitivity in Cancer.^15^ In addition, the DepMap and Cell Model Passports (Wellcome Sanger Institute, Hinxton, Cambridgeshire, UK)^16^ provided variant calls for a number of cell lines.

### Variant Drug Associations

The Genotype To Phenotype (G2P; Wellcome Sanger Institute) schema^17^ was designed to enable several different cancer knowledge-base resources to be aggregated into a coherent resource. The entries from these knowledge bases typically demarcate associations such as “the T41A mutation in *CTNNB1* causes sensitivity to imatinib.” With this resource, the BMEG has aggregated associations from six prominent cancer knowledge bases, including 50,000 associations vertices.

### Pathway Data

Pathway Commons (https://www.pathwaycommons.org/)^18^ aggregates, normalizes, and integrates data from 22 public pathway databases. At 1.5 million interactions and 400,000 detailed biochemical reactions, it is the largest curated pathway database available. It aggregates pathway relationships from Reactome (Wellcome Sanger Institute; European Molecular Biology Laboratory, Heidelberg, Germany),^19^ NCI Pathway Interaction Database (National Cancer Institute),^20^ PhosphoSitePlus (Cell Signaling Technology, Danvers, MA),^21^ HumanCyc (SRI International, Menlo Park, CA),^22^ PANTHER Pathway,^23^ MSigDB (Broad Institute, Massachusetts Institute of Technology, Cambridge, MA),^24^ Recon X,^25^ Comparative Toxicogenomics Database (North Carolina State University, Raleigh, NC),^26^ KEGG Pathway (Kyoto University, Kyoto, Japan),^27^ Integrating Network Objects with Hierarchies,^28^ NetPath (SolarWinds Worldwide, Austin, TX),^29^ and WikiPathways.^30^ Once loaded into the graph, these resources provided approximately 2 million vertices that could be queried by the user.

### Reference Data

The BMEG uses Ensembl identifiers^31^ as a global identifier to unite various genomic components present across the ingested biologic reference data and experimental results. The genomic annotations from Ensembl were modeled into the graph to provide a consistent chromosomal coordinate system for any sequence-level sample information. Part of the import pipeline includes annotating sample variants using Variant Effect Predictor^32^ to connect them to gene, transcript, and exon data from Ensembl. These, in turn, link to Protein and Pfam (protein family)^33^ assignments, as well as Gene Ontology^34^ functional annotations.

### Queries Using a Graph Language

To enable various analytical queries and provide a framework for analysts to build custom queries, we developed the Graph Integration Platform (GRIP) to design queries to use the BMEG. GRIP stores multiple forms of data and has the ability to hold thousands of data elements per vertex and per edge of the graph. This allows it to store sparse relationship data, such as pathways and ontologies, as well as dense matrix-formatted data, such as expression levels for thousands of genes across hundreds of samples.

The query language implements most operations needed for subgraph selection, as well as aggregation of features. A general purpose end point places more emphasis on the client side, building smart queries to obtain the data they need rather than having custom server-side components provide specialized facets. Because of this, clients can easily create new queries, unanticipated by the server developers, that still have the correct desired effect. The API is available via Python (Python Software Foundation, Wilmington, DE), Javascript (PluralSight, Farmington, UT), and R (R Foundation, https://www.r-project.org/) clients.

## RESULTS

To test the utility of the BMEG and its query engine, we have crafted example queries that traverse different parts of the graph, to demonstrate how the system can quickly provide an analyst with connected data. Although it would be possible for an analyst to find the solutions to the following exercises without using the BMEG, the analyst would need to download and merge data from multiple different repositories such as from the TCGA’s GDC system, somatic mutations predicted from Broad Institute’s CCLE collection and the TCGA’s MC3 variant-calling project, seven different somatic variant-to-phenotype association catalogs, PubChem for the names and modes of action of molecular compounds, pathway gene sets from Pathway Commons, and three different drug-response databases. Thus, the benefits of ingesting all into a uniform graph data structure should provide a more seamless presentation that users will find easier to use once the API becomes familiar.

**FIG 2.**
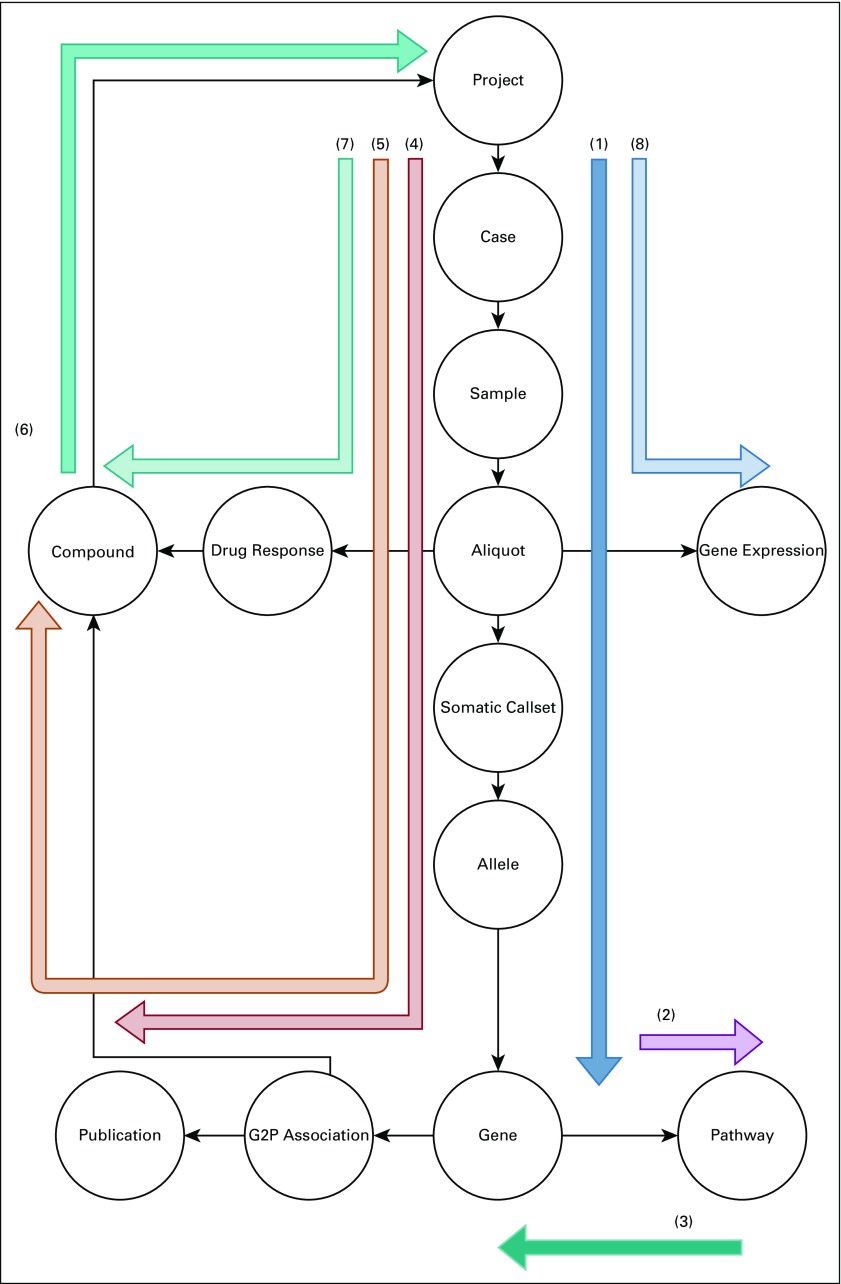
Example queries. A diagram showing how each of the different queries described in this article traverse the graph. Each separate query is labeled by the example number in the text.

The GRIP Query Language is a traversal-based graph-selection language inspired by Gremlin.^35^ The user describes a series of steps that will be undertaken by a “traveler.” An example traversal would start on a vertex with label *Project*, move to edges labeled *samples*, then move along edges labeled *aliquots*. The engine then scans the graph for all valid paths that can be completed given the instructions. Each of the traversal instructions is based on the graph schema seen in Figure 1. The commands are written using the Python version of the client, but they could be executed similarly in R or Javascript. These queries can be visualized as paths traversed through the BMEG graph (Fig 2).

We begin with an example that counts the mutations per gene in a cancer cohort. This is a useful statistic to gauge one aspect of whether a gene may be a driver of progression, as evidenced by its prevalence in a subpopulation (in this case, breast cancer). As seen in example 1 in the following section, the query starts on a breast cancer project node (TCGA-BRCA) then traverses to the *Case*, *Sample*, and *Aliquot* nodes while filtering out any data properties that do not belong to the previous node information. As it passes the *Sample* node, it filters for tumor samples. Once on the *Aliquot* node, it continues to the *SomaticCallset*, which represents sets of variants produced by a single mutation calling analysis. The traversal then identifies the edges that connect the *SomaticCallset* to different alleles, this time using the *outE* command to land on the edge rather than the destination vertex. With the gene identifier in hand, it then uses the *aggregate* method to count the various terms that occur in the *ensembl_gene* field.

### Example 1: Count Mutations per Gene in Breast Cancer

#### Query.

For example 1, the query is written as follows:

- G.query().V(“Project:TCGA-BRCA”)
- .out(“cases”).out(“samples”)
- .has(gripql.eq(“gdc_attributes.sample_type”, “Primary Tumor”))
- .out(“aliquots”).out(“somatic_callsets”).outE(“alleles”)
- .has(gripql.contains(“methods”, “MUTECT”))
- .aggregate(gripql.term(“geneCount”, “ensembl_gene”))

#### Result.

The result lists the number of variant alleles found for each gene. The example accesses data from the GDC as well as the MC3 somatic variant call set:

- ENSG00000155657 (n = 416)
- ENSG00000121879 (n = 401)
- ENSG00000141510 (n = 300)
- ENSG00000181143 (n = 193)
- ENSG00000198626 (n = 113)

### Example 2: Identify Pathways Containing Mutated Genes

There may be a theme among the most frequently mutated genes in breast cancer. The next two example queries address this by finding the most frequently affected pathways. First, example 2 identifies pathways containing mutated genes. Example 3 tallies the number of genes per pathway. To identify the pathways involved in mutations, the example derives a list of all mutated genes, finds their associated pathways, and retrieves the tuples of every gene-pathway pair, using the *as_* command (the underscore is added to avoid clashing with the Python-reserved word *as*) to store the gene and then using the *render* function to display only the needed data.

#### Query.

- G.query().V(genes).as_(“gene”)
- .out(“pathways”).render([“$gene._gid”, “$._gid”])

#### Result.

The result returns all the pathways for which each gene is a member. This query uses data extracted from Ensembl and Pathway Commons.

- ENSG00000000419 (pathwaycommons.org/pc11/Pathway_6bf6d39c0284b6...)
- ENSG00000000938 (identifiers.org/reactome/*R*-HSA-432142)
- ENSG00000000971 (identifiers.org/reactome/*R*-HSA-977606)
- ENSG00000001036 (pathwaycommons.org/pc11/Pathway_4b5817426aa06d...)

### Example 3: Determine the Number of Mutations in Each Pathway

Continuing in this investigation to derive the most affected pathways, the next step is to aggregate the mutations per pathway and then sum them. To do this, the preceding listed information can be combined with the previously found result tabulating the mutations per pathway. To sum all the mutations in all the genes in a particular pathway, the traversal starts on the *Pathway* vertex marked for later retrieval using the *as_* command. Once the traveler has split and moved out to the multiple child *Gene* vertices, the *select* command recalls the stored pathway vertex and moves the traveler back. At this point, an *aggregation* is called to count the number of travelers on each *Pathway* vertex.

#### Query.

- G.query().V().hasLabel(“Pathway”).as_(“pathway”)
- .out(“genes”).select(“pathway”)
- .aggregate(gripql.term(“pathwayGeneCount”, “_gid”))

#### Result.

The result lists the number of mutations for each pathway found.

- identifiers.org/reactome/*R*-HSA-191273 (n = 439)
- identifiers.org/reactome/*R*-HSA-381753 (n = 393)
- identifiers.org/reactome/*R*-HSA-212436 (n = 341)
- pathwaycommons.org/pc11/Pathway_4b5817426aa06d... (n = 340)

### Example 4: Find Publications Relevant to Phenotypic Consequences of Mutations

A biologist may wish to find evidence in the literature for any known phenotypic consequences of a collection of mutations, providing clues about the mechanisms involved in carcinogenesis. To this end, example 4 shows how the mutations found in the Breast Cancer Carcinoma (BRCA) cohort are linked to publications referenced by the G2P associations. In this use case, the aggregate method is called on the *_gid* variable, which represents a unique global identifier for each vertex.

#### Query.

- G.query().V(“Project:TCGA-BRCA”)
- .out(“cases”).out(“samples”)
- .has(gripql.eq(“gdc_attributes.sample_type”, “Primary Tumor”))
- .out(“aliquots”).out(“somatic_callsets”).out(“alleles”)
- .out(“g2p_associations”).out(“publications”)
- .aggregate(gripql.term(“pub”, “_gid”))

#### Result.

The result returns a list of the number of mutations for all genes connected in each of the returned papers. This query connects data from the GDC, the knowledge bases imported from the G2P project, and PubMed.

- Publication:ncbi.nlm.nih.gov/pubmed/27269946 (n = 1,033)
- Publication:ncbi.nlm.nih.gov/pubmed/27174596 (n = 1,029)
- Publication:ncbi.nlm.nih.gov/pubmed/19223544 (n = 858)
- Publication:ncbi.nlm.nih.gov/pubmed/20619739 (n = 664)

### Example 5: Find Drugs Described in the Literature to Treat Phenotypes Linked to Mutations

The phenotypes in the G2P associations, linked to the collected breast cancer mutations, may be associated with drugs that treat specific conditions. Example 5 defines a traversal of the graph to uncover compounds linked to phenotypes on the basis of specific alleles. The traversal is much like the one illustrated in example 4; however, it also includes a *distinct* operation to identify unique pairs of cases and compounds. If there are multiple known association records from different publications and these publications link one allele to the same drug-response phenotype, then only one relationship will be noted per patient.

#### Query.

- G.query().V(“Project:TCGA-BRCA”)
- .out(“cases”).as_(“case”).out(“samples”)
- .has(gripql.eq(“gdc_attributes.sample_type”,“Primary Tumor”))
- .out(“aliquots”).out(“somatic_callsets”).out(“alleles”)
- .out(“g2p_associations”).out(“compounds”)
- .distinct([“$case._gid”, “_gid”])
- .aggregate(gripql.term(“compound”, “_gid”))

#### Result.

The result lists the number of times compounds were associated to mutations in patients. This query uses data in the graph derived from the GDC, the MC3 callset, the G2P knowledge bases and PubChem.

- Compound:CID104741 (n = 345)
- Compound:CID11717001 (n = 340)
- Compound:CID56649450 (n = 327)
- Compound:NO_ONTOLOGY:CID24989044 (n = 313)

### Example 6: Find Drugs Tested in Breast Cancer Cell Lines

Taking the analysis one step further, the next two examples identify drugs proven effective against breast cancer cell lines as determined in the CTRP project. Example 6 identifies those compounds that have been tested in breast cancer cell lines as part of the CTRP project. The query uses the drugs found in example 5 through a list named *compounds* as a starting point.

#### Query.

- G.query().V(compounds).as_(“compound”).out(“projects”)
- .has(gripql.eq(“project_id”, “CTRP_Breast_Cancer”))
- .select(“compound”).render([“_gid”, “synonym”])

#### Result.

The result lists those drugs that were profiled in the CTRP effort. For example, at the top of the list, one finds that the compound fulvestrant has been tested against breast cancer cell lines in the CTRP project.

- Compound:CID104741: FULVESTRANT
- Compound:CID11717001: CHEMBL525191
- Compound:CID17755052: PICTILISIB
- Compound:CID24964624: CHEMBL1079175
- Compound:CID42611257: VEMURAFENIB
- Compound:CID56649450: ALPELISIB

### Example 7: Find the Sensitivity of Breast Cancer Cell Lines to a Drug

To get a sense of the effectiveness of each of these drugs, a natural extension of this line of inquiry is to find out how sensitive the cells are to them. The EC_50_ measures the concentration achieving a response midway between the baseline and maximum when cells are exposed to a drug. It is a widely used measure of sensitivity (although a measure, GR_50_, that factors in growth rate, has been shown to be more useful) and available for compounds tested in the CTRP project. To this end, example 7 searches for the EC_50_ values for the breast cancer cell lines tested against fulvestrant.

This query includes a call to the *render* method, which shapes the output into a custom JavaScript Object Notation structure (JSON). In this case, it forms a tuple with the stored sample identifier and EC_50_ value. The list of tuples returned by the client can then be passed directly into a Pandas DataFrame.^36^

#### Query.

- G.query().V(“Program:CTRP”).out(“projects”)
- .out(“cases”).out(“samples”).as_(“sample”)
- .out(“aliquots”).out(“drug_response”).as_(“response”)
- .out(“compounds”).hasId(“Compound:CID104741”)
- .render([“$sample._gid”,”$response.submitter_compound_id”,”$response.ec50”])

#### Result.

The result lists the EC_50_ values for each of the *BRCA* cell lines to fulvestrant, connecting the data from CTRP to PubChem entries.

- Sample:CTRP:ACH-000937: fulvestrant (n = 3.075000e−01)
- Sample:CTRP:ACH-000076: fulvestrant (n = 2.317000e−02)
- Sample:CTRP:ACH-000983: fulvestrant (n = 3.114000e−05)
- Sample:CTRP:ACH-000045: fulvestrant (n = 3.055000e−01)

### Example 8: Find Gene Expression Data Linked to Cell Lines

Drug response data often are not available for patient samples; thus, machine-learning methods that can use more widely available data, such as gene expression data from RNA sequencing, to predict drug response are highly promising. Example 8 illustrates how associated transcriptomic data can be obtained for the cell lines collected in the previous steps. There is no RNA sequencing available from the CTRP project; however, many of the cell lines were assayed as part of the complementary CCLE project. To identify these samples, example 8 follows the edge connecting the list named *samples* found in example 7 to their parent cases. It then follows the *same_as* edge to identify *Case* vertices in other projects that have overlapping identifiers, and then follows the tree down to the *GeneExpression* node to obtain the expression values. Again, the example uses the *render* function to return properly formatted data structures that can be passed directly into Pandas.

#### Query.

- G.query().V(samples).as_(“sample”)
- .out(“case”).out(“same_as”)
- .out(“samples”).out(“aliquots”).out(“gene_expressions”).as_(“exp”)
- .render([“$sample._gid”, “$exp._data.values”])

#### Result.

The resulting matrix (Table 1) lists the expression values of each gene across cell lines with variants in CTRP and RNA in CCLE. The matrix can be used to develop transcriptome-based drug-response prediction models.^37,38^

**TABLE 1.**
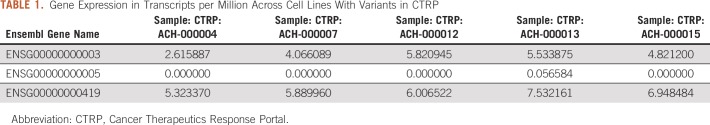
Gene Expression in Transcripts per Million Across Cell Lines With Variants in CTRP

##### Data Releases.

**FIG 3.**
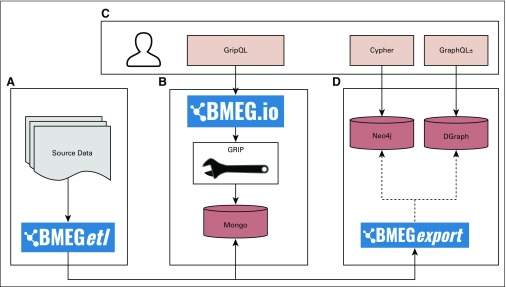
BioMedical Evidence Graph (BMEG) architecture diagram. (A) The Extract Transform Load (ETL) processes used to build the graph. (B) The database and query engine used to power the bmeg.io site. (C) The different client-side options for communicating with the system. (D) Graph engines that can be used with the BMEG export code to move the BMEG data to other graph databases. GripQL, Graph Integration Platform query language.

The BMEG resource was designed to be portable and open, with multiple ways to access the data (Fig 3). The graph query engine that runs the system is open source and easy to install, and all the compiled source files are made available for bulk download. This will allow other researchers to build on our existing system and to reuse the collected data. We provide translations of the BMEG to make it compatible with a number of different query engines. Part of the BMEG toolkit is a set of scripts to translate the data set and load it into other graph database systems, including Neo4J (San Mateo, CA) and Dgraph (Dgraph Labs, San Francisco, CA).

## DISCUSSION

Recently, several graph-based data integration projects have appeared, including biograkn,^39^ Biograph,^40^ Bio4j (discontinued),^41^ Bio2RDF,^42^ and Hetionet.^43^ Many of these systems were built to aggregate pathway and link genotype to phenotype. The BMEG holds genomic, transcriptomic, and phenotypic data from cancer cases, as well as from cell-line samples, pathway data, genomic descriptions, and extractions from genome-variant knowledge bases. The system includes unit tests composed of built-in Python conversion code, implemented in a Travis continuous integration facility, for technical validation to ensure data are copied and represented accurately. The unique accumulation of various high-quality data types differentiates the BMEG from other data systems. As demonstrated in the example queries, data interrogation can traverse sample mutations, pathway descriptions, knowledge bases, and drug-response data, all within a few lines of query code.

The fundamental idea of the BMEG is to define a connected data set to enable many possible investigations without the effort needed to collect, normalize, and merge information across disparate systems, thus saving time and effort to focus on research questions rather than data wrangling. Adopting systems like the BMEG will drive analyses that can tap a wide range of sample data, with structured annotations, allowing for a number of feature and prediction label combinations for machine-learning applications to support new pattern discovery.

## Figures and Tables

**TABLE A1.**
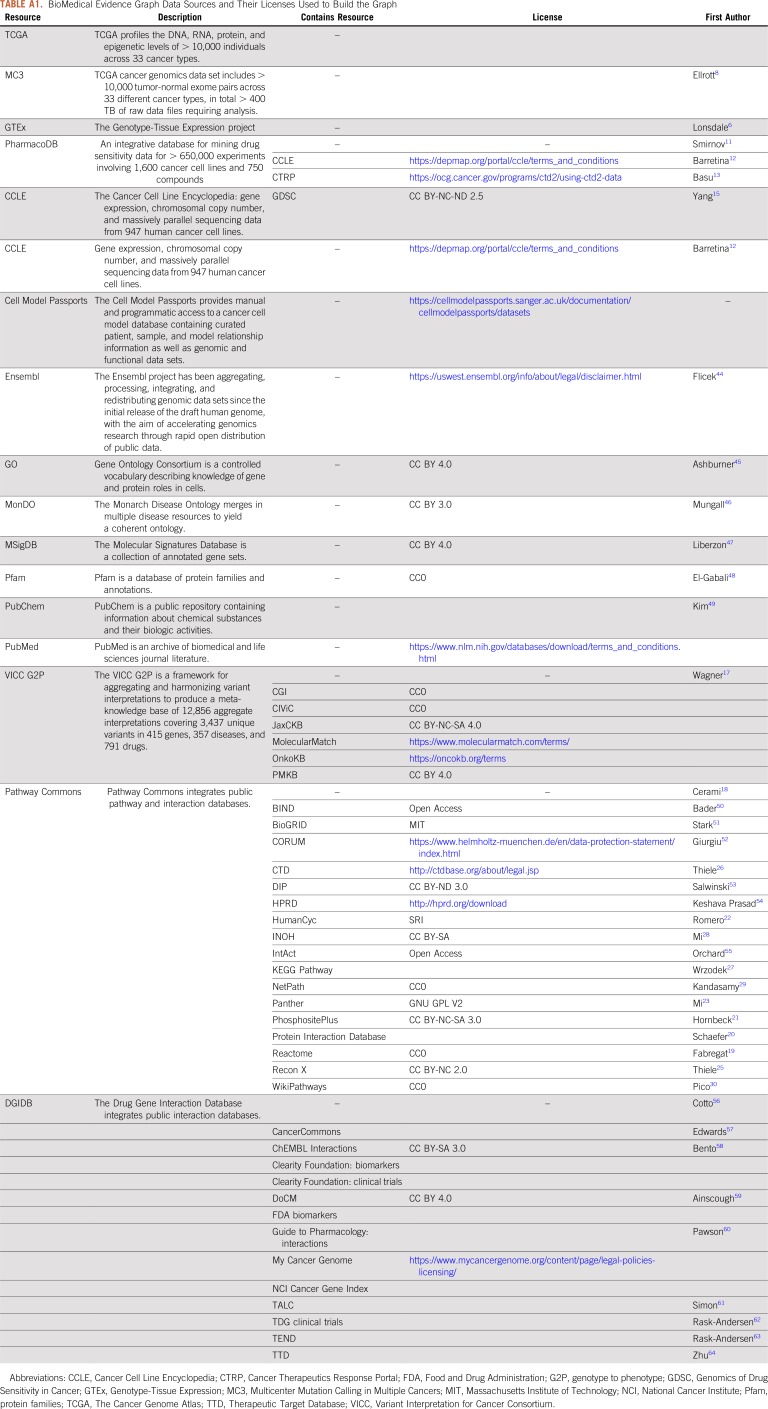
BioMedical Evidence Graph Data Sources and Their Licenses Used to Build the Graph

## References

[1] Yoon B-H Kim S-K Kim S-Y Use of graph database for the integration of heterogeneous biological data Genomics Inform 15 19 27 2017 28416946 10.5808/GI.2017.15.1.19 PMC5389944

[2] Have CT Jensen LJ Are graph databases ready for bioinformatics? Bioinformatics 29 3107 3108 2013 24135261 10.1093/bioinformatics/btt549 PMC3842757

[3] Lysenko A Roznovăţ IA Saqi M et al Representing and querying disease networks using graph databases BioData Min 9 23 2016 27462371 10.1186/s13040-016-0102-8 PMC4960687

[4] Ugander J Karrer B Backstrom L et alThe anatomy of the Facebook Social Graph. arXiv.1111.4503 [cs.SI]2011 http://arxiv.org/abs/1111.4503

[5] Tatlow PJ Piccolo SR A cloud-based workflow to quantify transcript-expression levels in public cancer compendia Sci Rep 6 39259 2016 27982081 10.1038/srep39259 PMC5159871

[6] Lonsdale J, Thomas J, Salvatore M, et al: The Genotype-Tissue Expression (GTEx) project. Nat Genet 45:580-585, 2013 10.1038/ng.2653 PMC4010069 23715323

[7] Boutros PC, Ewing AD, Ellrott K, et al: Global optimization of somatic variant identification in cancer genomes with a global community challenge. Nat Genet 46:318-319, 2014 10.1038/ng.2932 PMC4035501 24675517

[8] Ellrott K Bailey MH Saksena G et al Scalable open science approach for mutation calling of tumor exomes using multiple genomic pipelines Cell Syst 6 271 281.e7 2018 29596782 10.1016/j.cels.2018.03.002 PMC6075717

[9] Mermel CH Schumacher SE Hill B et al GISTIC2.0 facilitates sensitive and confident localization of the targets of focal somatic copy-number alteration in human cancers Genome Biol 12 R41 2011 21527027 10.1186/gb-2011-12-4-r41 PMC3218867

[10] Ghandi M Huang FW Jané-Valbuena J et al Next-generation characterization of the Cancer Cell Line Encyclopedia Nature 569 503 508 2019 31068700 10.1038/s41586-019-1186-3 PMC6697103

[11] Smirnov P Kofia V Maru A et al PharmacoDB: An integrative database for mining in vitro anticancer drug screening studies Nucleic Acids Res 46 D994 D1002 2018 30053271 10.1093/nar/gkx911 PMC5753377

[12] Barretina J, Caponigro G, Stransky N, et al: The Cancer Cell Line Encyclopedia enables predictive modelling of anticancer drug sensitivity. Nature 483:603-607, 2012 [Addendum: Nature 565:E5-E6, 2019] 10.1038/s41586-018-0722-x 30559381

[13] Basu A Bodycombe NE Cheah JH et al An interactive resource to identify cancer genetic and lineage dependencies targeted by small molecules Cell 154 1151 1161 2013 23993102 10.1016/j.cell.2013.08.003 PMC3954635

[14] Rees MG Seashore-Ludlow B Cheah JH et al Correlating chemical sensitivity and basal gene expression reveals mechanism of action Nat Chem Biol 12 109 116 2016 26656090 10.1038/nchembio.1986 PMC4718762

[15] Yang W Soares J Greninger P et al Genomics of Drug Sensitivity in Cancer (GDSC): A resource for therapeutic biomarker discovery in cancer cells Nucleic Acids Res 41 D955 D961 2013 23180760 10.1093/nar/gks1111 PMC3531057

[16] van der Meer D Barthorpe S Yang W et al Cell Model Passports-a hub for clinical, genetic and functional datasets of preclinical cancer models Nucleic Acids Res 47 D923 D929 2019 30260411 10.1093/nar/gky872 PMC6324059

[17] Wagner AH Walsh B Mayfield G et al A harmonized meta-knowledgebase of clinical interpretations of cancer genomic variants. bioRxiv 366856 2018 https://www.biorxiv.org/content/10.1101/366856v2

[18] Cerami EG Gross BE Demir E et al Pathway Commons, a web resource for biological pathway data Nucleic Acids Res 39 D685 D690 2011 21071392 10.1093/nar/gkq1039 PMC3013659

[19] Fabregat A Jupe S Matthews L et al The Reactome Pathway Knowledgebase Nucleic Acids Res 46 D649 D655 2018 29145629 10.1093/nar/gkx1132 PMC5753187

[20] Schaefer CF Anthony K Krupa S et al PID: the Pathway Interaction Database Nucleic Acids Res 37 D674 D679 2009 suppl 1 18832364 10.1093/nar/gkn653 PMC2686461

[21] Hornbeck PV Zhang B Murray B et al PhosphoSitePlus, 2014: Mutations, PTMs and recalibrations Nucleic Acids Res 43 D512 D520 2015 25514926 10.1093/nar/gku1267 PMC4383998

[22] Romero P Wagg J Green ML et al Computational prediction of human metabolic pathways from the complete human genome Genome Biol 6 R2 2005 15642094 10.1186/gb-2004-6-1-r2 PMC549063

[23] Mi H Huang X Muruganujan A et al PANTHER version 11: Expanded annotation data from Gene Ontology and Reactome pathways, and data analysis tool enhancements Nucleic Acids Res 45 D183 D189 2017 27899595 10.1093/nar/gkw1138 PMC5210595

[24] Subramanian A Tamayo P Mootha VK et al Gene set enrichment analysis: A knowledge-based approach for interpreting genome-wide expression profiles Proc Natl Acad Sci USA 102 15545 15550 2005 16199517 10.1073/pnas.0506580102 PMC1239896

[25] Thiele I Swainston N Fleming RMT et al A community-driven global reconstruction of human metabolism Nat Biotechnol 31 419 425 2013 23455439 10.1038/nbt.2488 PMC3856361

[26] Davis AP Grondin CJ Johnson RJ et al The Comparative Toxicogenomics Database: Update 2017 Nucleic Acids Res 45 D972 D978 2017 27651457 10.1093/nar/gkw838 PMC5210612

[27] Wrzodek C Büchel F Ruff M et al Precise generation of systems biology models from KEGG pathways BMC Syst Biol 7 15 2013 23433509 10.1186/1752-0509-7-15 PMC3623889

[28] Yamamoto S Sakai N Nakamura H et al INOH: Ontology-based highly structured database of signal transduction pathways Database (Oxford) 2011 bar052 2011 22120663 10.1093/database/bar052 PMC3225078

[29] Kandasamy K Mohan SS Raju R et al NetPath: A public resource of curated signal transduction pathways Genome Biol 11 R3 2010 20067622 10.1186/gb-2010-11-1-r3 PMC2847715

[30] Pico AR Kelder T van Iersel MP et al WikiPathways: Pathway editing for the people PLoS Biol 6 e184 2008 18651794 10.1371/journal.pbio.0060184 PMC2475545

[31] Hubbard T Barker D Birney E et al The Ensembl genome database project Nucleic Acids Res 30 38 41 2002 11752248 10.1093/nar/30.1.38 PMC99161

[32] McLaren W Gil L Hunt SE et al The Ensembl variant effect predictor Genome Biol 17 122 2016 27268795 10.1186/s13059-016-0974-4 PMC4893825

[33] Finn RD Bateman A Clements J et al Pfam: The protein families database Nucleic Acids Res 42 D1 D222 D230 2014 24288371 10.1093/nar/gkt1223 PMC3965110

[34] Carbon S, Mungall C: Gene Ontology Data Archive. 2018.

[35] Rodriguez MA: The Gremlin Graph Traversal Machine and Language. arXiv:1508.03843 [cs.DB]. 2015 http://arxiv.org/abs/1508.03843

[36] McKinney W Data structures for statistical computing in pythoninProceedings of the 9th Python in Science Conference Austin, TX 2010 51 56

[37] Sakellaropoulos T Vougas K Narang S et al A deep learning framework for predicting response to therapy in cancer Cell Rep 29 3367 3373.e4 2019 31825821 10.1016/j.celrep.2019.11.017

[38] Costello JC Heiser LM Georgii E et al A community effort to assess and improve drug sensitivity prediction algorithms Nat Biotechnol 32 1202 1212 2014 24880487 10.1038/nbt.2877 PMC4547623

[39] Messino A, Pribadi H, Stichbury J: BioGrakn: A knowledge graph-based semantic database for biomedical sciences, in Barolli K, Terzo O, eds: Complex, Intelligent, and Software Intensive Systems. New York, NY, Springer International Publishing, 2018:299-309

[40] Messina A Fiannaca A La Paglia L et al BioGraph: A web application and a graph database for querying and analyzing bioinformatics resources BMC Syst Biol 12 98 2018 30458802 10.1186/s12918-018-0616-4 PMC6245492

[41] Reference deleted

[42] Belleau F Nolin M-A Tourigny N et al Bio2RDF: Towards a mashup to build bioinformatics knowledge systems J Biomed Inform 41 706 716 2008 18472304 10.1016/j.jbi.2008.03.004

[43] Reference deleted

[44] Flicek P Amode MR Barrell D et al Ensembl 2011 Nucleic Acids Res 39 D800 D806 2011 21045057 10.1093/nar/gkq1064 PMC3013672

[45] Ashburner M Ball CA Blake JA et al Gene ontology: Tool for the unification of biology Nat Genet 25 25 29 2000 10802651 10.1038/75556 PMC3037419

[46] Mungall CJ McMurry JA Köhler S et al The Monarch Initiative: An integrative data and analytic platform connecting phenotypes to genotypes across species Nucleic Acids Res 45 D712 D722 2017 27899636 10.1093/nar/gkw1128 PMC5210586

[47] Liberzon A Subramanian A Pinchback R et al Molecular signatures database (MSigDB) 3.0 Bioinformatics 27 1739 1740 2011 21546393 10.1093/bioinformatics/btr260 PMC3106198

[48] El-Gebali S Mistry J Bateman A et al The Pfam protein families database in 2019 Nucleic Acids Res 47 D427 D432 2019 30357350 10.1093/nar/gky995 PMC6324024

[49] Kim S Thiessen PA Bolton EE et al PubChem substance and compound databases Nucleic Acids Res 44 D1202 D1213 2016 26400175 10.1093/nar/gkv951 PMC4702940

[50] Bader GD Betel D Hogue CWV BIND: The Biomolecular Interaction Network Database Nucleic Acids Res 31 248 250 2003 12519993 10.1093/nar/gkg056 PMC165503

[51] Stark C Breitkreutz B-J Reguly T et al BioGRID: A general repository for interaction datasets Nucleic Acids Res 34 D535 D539 2006 16381927 10.1093/nar/gkj109 PMC1347471

[52] Giurgiu M Reinhard J Brauner B et al CORUM: The comprehensive resource of mammalian protein complexes-2019 Nucleic Acids Res 47 D559 D563 2019 30357367 10.1093/nar/gky973 PMC6323970

[53] Salwinski L Miller CS Smith AJ et al The Database of Interacting Proteins: 2004 Update Nucleic Acids Res 32 D449 D451 2004 14681454 10.1093/nar/gkh086 PMC308820

[54] Keshava Prasad TS Goel R Kandasamy K et al Human Protein Reference Database–2009 update Nucleic Acids Res 37 D767 D772 2009 18988627 10.1093/nar/gkn892 PMC2686490

[55] Orchard S Ammari M Aranda B et al The MIntAct project–IntAct as a common curation platform for 11 molecular interaction databases Nucleic Acids Res 42 D358 D363 2014 24234451 10.1093/nar/gkt1115 PMC3965093

[56] Cotto KC Wagner AH Feng Y-Y et al DGIdb 3.0: A redesign and expansion of the drug-gene interaction database Nucleic Acids Res 46 D1068 D1073 2018 29156001 10.1093/nar/gkx1143 PMC5888642

[57] Edwards AM Isserlin R Bader GD et al Too many roads not taken Nature 470 163 165 2011 21307913 10.1038/470163a

[58] Bento AP Gaulton A Hersey A et al The ChEMBL bioactivity database: An update Nucleic Acids Res 42 D1083 D1090 2014 24214965 10.1093/nar/gkt1031 PMC3965067

[59] Ainscough BJ Griffith M Coffman AC et al DoCM: A database of curated mutations in cancer Nat Methods 13 806 807 2016 27684579 10.1038/nmeth.4000 PMC5317181

[60] Pawson AJ Sharman JL Benson HE et al The IUPHAR/BPS Guide to Pharmacology: An expert-driven knowledgebase of drug targets and their ligands Nucleic Acids Res 42 D1098 D1106 2014 24234439 10.1093/nar/gkt1143 PMC3965070

[61] Simon GR Somaiah N A tabulated summary of targeted and biologic therapies for non-small-cell lung cancer Clin Lung Cancer 15 21 51 2014 24377743 10.1016/j.cllc.2013.11.009

[62] Rask-Andersen M Masuram S Schiöth HB The druggable genome: Evaluation of drug targets in clinical trials suggests major shifts in molecular class and indication Annu Rev Pharmacol Toxicol 54 9 26 2014 24016212 10.1146/annurev-pharmtox-011613-135943

[63] Rask-Andersen M Almén MS Schiöth HB Trends in the exploitation of novel drug targets Nat Rev Drug Discov 10 579 590 2011 21804595 10.1038/nrd3478

[64] Zhu F Han B Kumar P et al Update of TTD: Therapeutic target database Nucleic Acids Res 38 D787 D791 2010 suppl 1 19933260 10.1093/nar/gkp1014 PMC2808971

